# Deep Eutectic Solvents as Catalysts for Cyclic Carbonates Synthesis from CO_2_ and Epoxides

**DOI:** 10.3390/molecules27249006

**Published:** 2022-12-17

**Authors:** Dorota Mańka, Agnieszka Siewniak

**Affiliations:** Department of Chemical Organic Technology and Petrochemistry, Faculty of Chemistry, Silesian University of Technology, Krzywoustego 4, 44-100 Gliwice, Poland

**Keywords:** cyclic carbonates, deep eutectic solvent, carbon dioxide, epoxide, cycloaddition of CO_2_

## Abstract

In recent years, the chemical industry has put emphasis on designing or modifying chemical processes that would increasingly meet the requirements of the adopted proecological sustainable development strategy and the principles of green chemistry. The development of cyclic carbonate synthesis from CO_2_ and epoxides undoubtedly follows this trend. First, it represents a significant improvement over the older glycol phosgenation method. Second, it uses renewable and naturally abundant carbon dioxide as a raw material. Third, the process is most often solvent-free. However, due to the low reactivity of carbon dioxide, the process of synthesising cyclic carbonates requires the use of a catalyst. The efforts of researchers are mainly focused on the search for new, effective catalysts that will enable this reaction to be carried out under mild conditions with high efficiency and selectivity. Recently, deep eutectic solvents (DES) have become the subject of interest as potential effective, cheap, and biodegradable catalysts for this process. The work presents an up-to-date overview of the method of cyclic carbonate synthesis from CO_2_ and epoxides with the use of DES as catalysts.

## 1. Introduction

**Figure 1:**
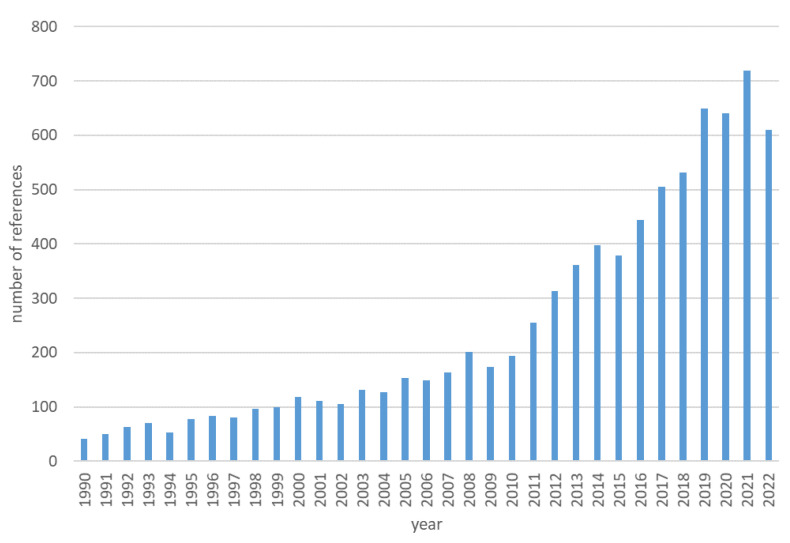
Number of publications on cyclic carbonates in the last 30 years (CAS SciFinder database).

Cyclic carbonates, e.g., propylene and ethylene carbonates, are important compounds in the chemical industry, and have attracted a lot of interest over the last decades (Figure 1) [1]. This is mostly due to their unique property sets: thermal stability, low toxicity and easy biodegradability. Therefore, they have a broad spectrum of applications, mainly as solvents in various products [2,3], e.g., in lithium-ion batteries, cleaning and degreasing agents [3], industrial lubricants [4] and fuel additives [5]. They are also used to produce monomers [4] and polymers such as polycarbonates [2], polyester resins, and polyurethanes [3]. Cyclic carbonates have applications in the production of pharmaceuticals, agrochemicals [2], and other fine chemicals [4]. They are also used as intermediates in many chemical processes, e.g., to produce linear dialkyl carbonates [6], and can undergo chemical reactions such as hydrogenation, transesterification and substitution [3,7].

Cyclic carbonates can be synthesized in many ways, for example from diols and phosgene, urea or carbon oxides [4]. There are also reports of methods using halo-alcohols [6] or olefins with CO_2_ [8]. Currently, processes utilizing carbon dioxide as feedstock are gaining the most interest. This is mainly due to the increasing impact of the environmental awareness related to the greenhouse effect, which is contributed to by the large amounts of CO_2_ released into the atmosphere [9]. It has become essential to capture and utilize already produced carbon dioxide. This abundant compound can be a valuable source of raw material for further syntheses in the chemical industry.

One of the most attractive approaches leading to the obtaining of cyclic carbonates is the catalytic reaction of CO_2_ with epoxides. This reaction has an atom economy of 100% and is a greener and safer alternative to the toxic phosgene route [10]. As the reaction of carbon dioxide and epoxide can only occur in the presence of a catalyst [11], many different catalytic systems have been developed for this synthesis. The most common catalysts are quaternary onium salts (including ammonium, phosphonium and imidazolium halides [10,12,13,14]), Lewis bases [10,15,16], *N*-heterocyclic carbenes [17], ionic liquids (ILs) [18,19,20,21], deep eutectic solvents (DES) [22,23,24], metal oxides (e.g., MgO, Nb_2_O_5_) [10,25], metal halides [10] and metal-organic complexes [10,26], including metal-organic frameworks [27].

Using deep eutectic solvents as catalysts for the cycloaddition of CO_2_ to epoxides is particularly interesting due to their characteristics and high catalytic activity [28]. They have properties similar to ionic liquids, such as being a liquid at room temperature, low volatility, tuneability, non-flammability, chemical and thermal stability, and being a good solvent. However, they have more advantages than ILs, such as lower price (resulting from cheaper substrates and also ease of preparation and storage), lower toxicity, and also good biodegradability and biocompatibility, which lead to fewer problems with waste disposal [29,30]. Deep eutectic solvents are defined as mixtures with a melting point significantly lower than the melting points of their constituents. They usually consist of two components—a hydrogen bond donor (HBD) and a hydrogen bond acceptor (HBA), although ternary mixtures are also possible [31,32]. They can often be easily prepared from natural and abundant components, such as dicarboxylic acids, urea, choline chloride (ChCl), polyols, sugars, or amino acids [28,33,34,35,36,37]. The application of DESs in the synthesis of cyclic carbonates is also beneficial due to the better solubility of CO_2_ in them compared to IL [38] and enhancement of the reaction rate by hydrogen bonds [39], which virtually create this kind of solvent [33]. In addition, homogeneous catalysts are often difficult to separate after the reaction, which is a significant drawback, especially in large-scale production, while deep eutectic solvents are often easy to separate and recycle and can be used in many consecutive reaction runs [40].

This article provides an overview of the up-to-date progress in DES-based catalytic systems for the preparation of cyclic carbonates from CO_2_ and epoxides. In this work, we present the most important, interesting and noted examples of cyclic carbonate synthesis to show the development of this issue over the last dozen or so years, and to present the potential of this method with its advantages and problems. We also indicate the main directions of development of the synthesis of cyclic carbonates involving DESs.

## 2. Deep Eutectic Solvents as Catalysts for Cyclic Carbonates Synthesis from Epoxides and CO_2_

### 2.1. General Information

Deep eutectic solvents generally consist of two compounds—a hydrogen bond donor and a hydrogen bond acceptor [31]. They interact with each other through hydrogen bonds and also through electrostatic and van der Waals forces. However, hydrogen bonds are the dominant interactions and they have the main influence on the properties of DESs [30]. Based on the character of the components of which DESs are comprised, several types of DES can be distinguished [29,31,41,42]: type I: quaternary salt + metal halide; type II: quaternary salt + metal halide hydrate; type III: quaternary salt + HBD; type IV: metal halide hydrate + HBD; and type V: non-ionic molecular HBAs + HBDs.

#### 2.1.1. DES Synthesis

The preparation of DESs is simple. Normally, it requires both compounds (HBA and HBD) to be mixed in an appropriate molar ratio, then heated to about 60–110 °C and stirred until a homogenous liquid is formed. Usually, it takes about an hour. The components are also often dried beforehand due to the influence of water on the properties of DESs, which will be discussed in more detail in Section 2.1.3. of this review [22,28,36,43].

#### 2.1.2. Mechanism of Cyclic Carbonate Synthesis from CO_2_ and Epoxides in the Presence of DES

**Figure 2:**
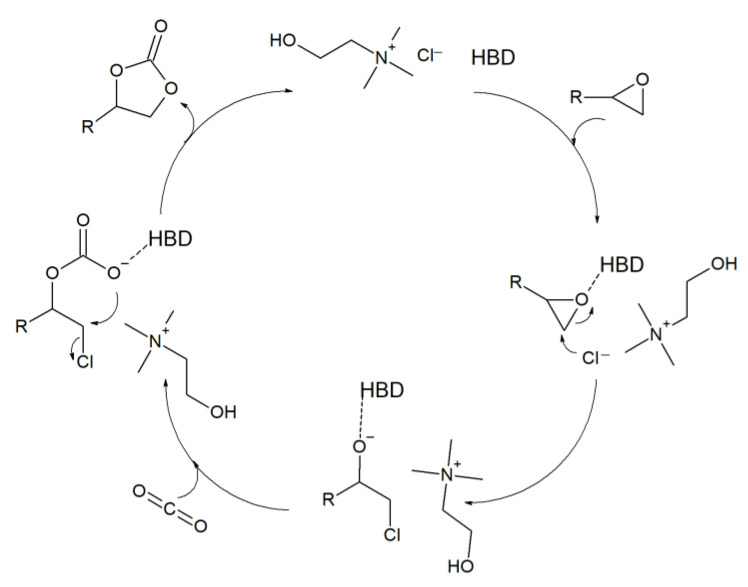
Proposed mechanism for cyclic carbonate synthesis catalyzed by a ChCl-based deep eutectic solvent [36].

The mechanism of CO_2_ cycloaddition to epoxides in the presence of a deep eutectic solvent as a catalyst, using the example of a choline chloride (ChCl)-based DES, is depicted in Figure 2 [36]. A hydrogen bond is created between the hydrogen bond donor and the oxygen of the epoxide, which activates the molecule and promotes the opening of its ring. This is continued by the attack of a nucleophile, which leads to the formation of an alkoxide intermediate. Then the CO_2_ molecule is incorporated, the ring closes back, forming the cyclic carbonate, and the catalyst is regenerated. Due to the formation of hydrogen bonds, the HBD here helps to stabilize the intermediates involved in the reaction.

#### 2.1.3. The Influence of Water

Deep eutectic solvents are usually hygroscopic substances, and they usually cannot be dried completely. Investigating the influence of water on DESs is important because it can interact with each component of such a solvent. The addition of water can break the hydrogen bonds existing between DES’s substrates. For example, water added to a ChCl:urea mixture reduces the strength of the hydrogen bonds between these two components and forms new ones between itself and urea. Even a very small amount of water added to DES can change its physical properties and the mixture becomes ternary [44].

#### 2.1.4. The Influence of Hydroxyl Groups, pK_a_ and Acidity of DES

The coupling of HBDs with organic salts is a common method used for developing new catalytic systems for the synthesis of cyclic carbonates. One of the most popular donor classes are compounds that have one or more hydroxyl groups, e.g., alcohols, polyalcohols, glycols or fluorinated alcohols and compounds with phenols and polyphenols. In the 2019 study carried out by Yingcharoen et al. [45], the question of whether the number of hydroxyl groups in HBD influences their performance in the synthesis of cyclic carbonates under ambient conditions was investigated. They did not observe a correlation between the number of hydroxyl groups and HBD activity. However, it transpired that in some cases the catalytic activity can be tuned by the presence of additional hydroxyl groups as long as their presence does not influence the pK_a_ of the donor. The researchers also showed that an important correlation occurs between the catalytic activity of HBD and the pK_a_ value of its most acidic proton. The activity of HBD increases with the increase in pK_a_ in the range of 3 to 11, while pK_a_ values over 12 have the opposite effect. Hydroxyl donors with a pK_a_ between 9 and 11 give the best results in obtaining cyclic carbonates from epoxides and CO_2_.

The influence of DES acidity on its activity in the synthesis of cyclic carbonates was investigated by Wang et al. [32]. It was demonstrated that deep eutectic solvents with higher acidity show stronger catalytic activity in the reaction of CO_2_ with epoxides, leading to the formation of cyclic carbonates. The description of these studies has been elaborated in Section 2.2. of this paper.

#### 2.1.5. The Influence of Temperature, Pressure and Reaction Time

An increase in temperature and pressure within a certain range usually has a beneficial effect on the reaction of CO_2_ with epoxide. In a paper from 2017, the influence of temperature and pressure used during the reaction of cycloaddition of CO_2_ to propylene oxide (PO) epoxides in the presence of DES composed of L-proline (L-Pro) and propanedioic acid (PA), was investigated [33]. It turned out that increasing the reaction temperature has a positive effect on the yield, but only in a certain temperature range. When the temperature exceeded 150 °C, the yield of the product decreased. This dependency is probably due to the intensification of side reactions that occur under such conditions. A similar phenomenon was observed in [28]. However, in the work of Liu et al., a decrease in product yield was observed above 120 °C [46].

As for the pressure, in the reaction of CO_2_ and PO catalyzed by L-Pro/PA, increasing it up to 1.2 MPa had a positive effect on the yield of propylene carbonate (PC) [33]. However, a further rise in pressure caused a drop in cyclic carbonate yield. Similar results were observed in other works [28,42,46], although the pressure limit was different and depended on the reaction system. For the reaction involving DES composed of choline chloride and PEG, the product yield occurred after exceeding the pressure of 0.8 MPa [28]. It is worth noting that the CO_2_ solubility in DES increases with higher pressure, which results in a better reaction outcome, but only up to a certain value. The further increase causes the dilution of epoxide with carbon dioxide, which has a negative effect on the reaction.

The reaction time depends mainly on the reaction system and the reaction conditions used. Usually, increasing the reaction time has a positive effect on the outcome. In the reaction of CO_2_ and PO in the presence of a L-Pro/PA catalyst, the product yield increased when the time was extended to 5 h, but it started declining when the time was longer [33]. Similarly to using a too-high temperature, carrying out the reaction for too long causes a rise in side reactions.

During the process of synthesizing cyclic carbonates from CO_2_ and epoxides, side reactions such as isomerization and dimerization of epoxides and hydrolysis leading to the formation of appropriate glycols may occur [36,46,47,48].

#### 2.1.6. The Influence of Substrates

Many of the developed DES were tested in the synthesis of a wide variety of cyclic carbonates from CO_2_ and various epoxides. For example, Liu et al. used propylene oxide, 1,2-epoxybutane, 1,2-epoxyhexane, epichlorohydrin, epibromohydrin, styrene oxide and cyclohexene oxide to synthesize the corresponding cyclic carbonates [40,43]. The highest yields were obtained for epichlorohydrin (99%), epibromohydrin (97%), and propylene oxide (96%), while the lowest yield was obtained for cyclohexene oxide (19%) [40]. In contrast, styrene carbonate was obtained with a yield of 87%. Similarly, in the work of Lü et al., the yield of the corresponding cyclic carbonates was the highest for propylene oxide (98.6%) and the lowest for cyclohexene oxide (2.5%) [33]. Based on the research, it can be concluded that terminal linear aliphatic epoxides without steric hindrance and epoxides with electron-withdrawing substituents, such as epichlorohydrin, allow obtaining cyclic carbonates with high yields. In epichlorohydrin, the presence of a chlorine atom in its structure makes the exposed carbon atom in the epoxide ring more susceptible to nucleophile attack [40]. In contrast, the use of 1,2-epoxycyclohexane with a steric hindrance resulting from the presence of two rings in its molecule reduces its reactivity [24,40].

#### 2.1.7. Recycling of the DES-Based Catalysts

The possibility of separating and reusing the catalyst is one of the key factors in developing new synthetic methods for industrial applications. Therefore, in most works on the use of DES as catalysts for the synthesis of cyclic carbonates, an attempt was made to recycle them. Extraction was the most commonly used method of catalyst separation. Vagnoni et al. added ethyl acetate with a small amount of water to the post-reaction mixture, resulting in two phases: organic, containing the product and unreacted substrate, and water with a dissolved catalyst [36]. After phase separation, the DES catalyst was recovered by water distillation and used directly in the next process. DES retained its activity in three consecutive cycles. The same method of catalyst separation was used, for example, by Yang et al. [23]. In other works, ethyl acetate [24] or MTBE alone [40] or a mixture of ethyl acetate and ether [49] were used for extraction. Wu et al., in turn, separated the mixture after the reaction, distilled off the cyclic carbonate, and the remaining catalyst was used directly for the next cycle [28]. In a similar manner, the catalyst was recovered by Lü et al. [33]. In the case of a supported DES (e.g., on molecular sieves [50] or lignin [51]), the catalyst can be separated by simple filtration.

### 2.2. DES-Based Catalytic Systems Used in the Synthesis of Cyclic Carbonates—An Overview

One of the first attempts to use deep eutectic solvents in the synthesis of cyclic carbonates from CO_2_ and epoxides was reported by Zhu et al. [50]. They applied a mixture of ChCl:urea in a 1:2 molar ratio as a catalyst for the reaction of CO_2_ and propylene oxide leading to propylene carbonate. The process was carried out in a 1.5 to 1.87 molar ratio of CO_2_ to epoxide, at 110 °C and for 10 h. The new catalyst transpired to be not only biodegradable, but also active and selective in the studied reaction. The yield of propylene carbonate was higher (99%) than in the reaction with choline chloride alone (85%). However, when the DES was immobilized on molecular sieves (Si/Al 1:1, pores size 6.7 nm), the time was decreased to only 4 h with unchanged yield. The authors assume that the synergistic effect of the anion and the cation of ChCl, and the interaction of urea with Cl^−^ have a major impact on the activity and selectivity of DES. The developed catalytic system was used for the reaction of other epoxides (such as epichlorohydrin, styrene oxide, cyclohexene oxide and phenoxyoxirane) with CO_2_, affording the corresponding cyclic carbonates in yields ranging from 80% to 99%. After the reaction, the catalyst constitutes a separate phase and can be easily regenerated and reused up to at least five times without a significant loss in activity.

**Figure 3:**
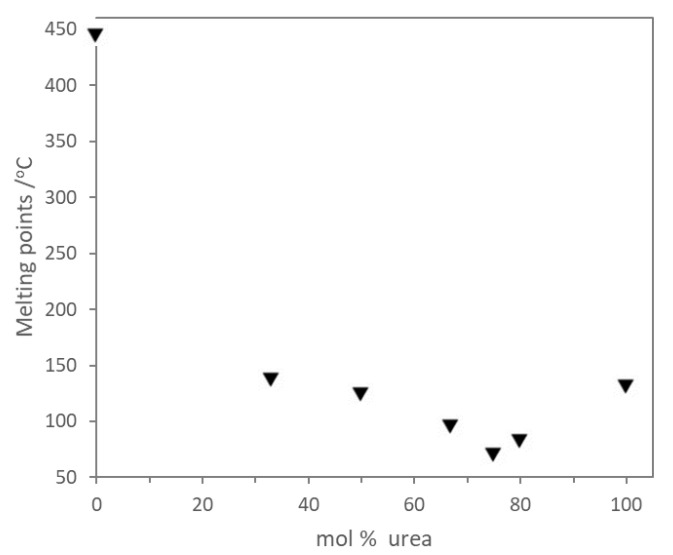
Dependence of DES ([urea-Zn]I_2_) melting points on DES composition. Reprinted with permission from Ref. [46].

In 2016, Liu et al. developed a catalyst consisting of urea and different zinc halides, for the synthesis of cyclic carbonates from epoxides and CO_2_ [46]. The obtained eutectic-based ionic liquids exhibited lower melting points than the melting points of the individual components, which is a characteristic feature of DESs. In the case of the urea:ZnI_2_ mixture, the lowest temperature was achieved for a molar ratio of 3:1 (Figure 3).

Combinations of zinc chloride, bromide and iodide with urea were tested. At a temperature of 120 °C, CO_2_ pressure of 1.0 MPa and after 2 h, the selectivity to propylene carbonate always reached 98%, but only in the presence of the [urea-Zn]I_2_ catalyst, the yield was also significant (84%). When the time was prolonged to 3 h, the yield was even higher (95%). It is worth noting that if ZnI_2_ and urea were introduced separately into the reaction, a lower yield was obtained (72%). Other epoxides were also tested in this reaction and yields ranged from 81 to 97%. After the reaction, the catalyst could be recycled by separating it through centrifugation, washing and drying, and used for four consecutive runs, in which it retained its selectivity but the yield slowly diminished. The catalyst was efficient, easy to prepare, reusable, and in comparison to pure ZnI_2_, [urea-Zn]I_2_ DES was less sensitive to moisture. However, the main drawbacks are the separation of the product requiring the use of dichloromethane and the necessity of applying relatively high temperatures and pressure.

In 2016, Saptal and Bhanage introduced an efficient catalytic system consisting of an ionic liquid and a quaternary ammonium salt for the synthesis of cyclic carbonates [52]. The tested ionic liquids were composed of amino acids (such as proline [Pro], glycine [Gly], alanine [Ala], or histidine [His]) and choline chloride, while the ammonium salts were tetrabutylammonium bromide (TBAB) or tetrabutylammonium iodide (TBAI). The researchers chose ILs to be composed of inexpensive, natural, biodegradable, and non-toxic compounds, hence the use of amino acids and choline chloride. In addition, the presence of HBDs in ILs in combination with amine groups can form an active catalyst for the synthesis of cyclic carbonates. It was also assumed that the amine groups of amino acids could improve the absorption of CO_2_ and thus the reaction parameters. The model reaction in this study was the cycloaddition of CO_2_ to epichlorohydrin (EP) under ambient conditions—at room temperature and under atmospheric pressure. IL ([Ch][Pro]) alone turned out to be ineffective and no cyclic carbonate was formed. However, when it was mixed with TBAB or TBAI, the catalyst gave a good performance—the selectivity reached 99% and the conversion was around 60%. The authors observed the formation of low-viscosity DES from the ionic liquid [Ch][Pro] and quaternary ammonium salts. If the pressure was increased to 1 MPa, the conversion was 73%, while after an additional increase in temperature to 70 °C, the conversion reached 98% for the [Ch][His]/TBAI catalytic system. The influence of potassium iodide instead of quaternary ammonium salt was also investigated—it gave worse results than TBAI but similar results to TBAB. After the reaction, the catalyst was regenerated and reused in the next 5 runs under two different pressures. When the reactions were carried out under atmospheric pressure, the cyclic carbonate yield diminished with each consecutive run, while for the reactions at 1 MPa, the yield decreased only slightly. It was suggested that this may be the influence of the reaction time on the activity of the catalyst—at the lower pressure the reaction time was 30 h, and at the higher pressure only 2 h. The applied catalytic system gave satisfying results under ambient conditions, but required a very long reaction time. However, when the pressure and temperature were increased, the outcome was excellent.

Deep eutectic solvents based on amino acids as HBAs (such as alanine, glycine, L-proline) and dicarboxylic acids as HBDs (oxalic acid and propanedioic acid, succinic acid) in cycloaddition reactions of CO_2_ to propylene oxide were investigated [33]. The DES based on L-proline and propanedioic acid was the most active; however, to obtain satisfactory results, it was necessary to use zinc bromide as a co-catalyst. The cyclic carbonates were obtained in good yields (56.6–98%) and selectivity (99%) at 150 °C and under a pressure of 1.2 MPa after 5 h. The authors emphasized that DES activity was influenced by the length of the carbon chain of the dicarboxylic acid. The chain length determines the strength of the hydrogen bond between DES and epoxide. Among the tested acids, the most preferred was propanedioic acid. Acids with shorter or longer chains formed too strong or too weak H-bonds. The DES and ZnBr_2_ could be recovered after distilling off the cyclic carbonate, and could be used at least nine times without loss in its activity. The main drawback of this catalytic system is the need to use toxic ZnBr_2_ as a co-catalyst, which has a negative impact on the environment. For this reason, the process cannot be considered fully “green” [28]. Catalyst separation after the reaction can also be problematic as the distillation process is energy-intensive, making the process not environmentally friendly and generating additional costs.

**Figure 4:**
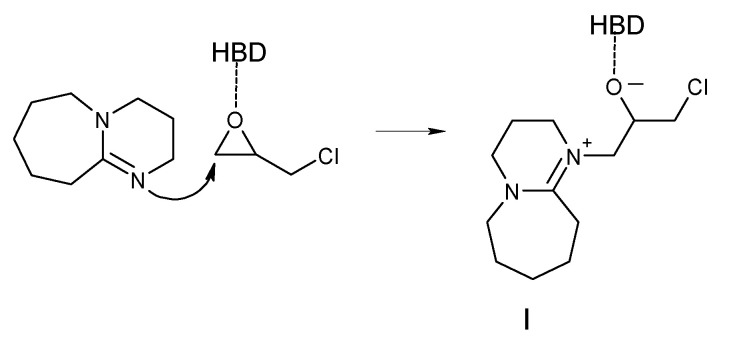
Activation of the epoxide by DES composed of DBU as a HBA proposed by García-Argüelles et al. [53].

In 2017, García-Argüelles et al. conducted a reaction of carbon dioxide with epoxides using deep eutectic solvents based on superbases [53]. The superbases alone have already been applied in this process with good results [54]. In addition, superbase-based DESs show the ability to absorb CO_2_ [55]. García-Argüelles et al. [53] used superbases as hydrogen bond acceptors (1,8-diazabicyclo[5.4.0]undec-7-ene (DBU) and 1,5,7-triazabicyclo[4.4.0]dec-5-ene (TBD)) and combined them with mono- and polyalcohols as hydrogen bond donors (ethylene glycol, methyldiethanolamine and benzyl alcohol). The tested reaction was the cycloaddition of CO_2_ to epichlorohydrin. The highest yield (98%) and selectivity (98%) were achieved using DES consisting of TBD and benzyl alcohol in a molar ratio of 1:1. The reaction time was only 2 h and the process was carried out at a temperature of 100 °C and a pressure of 0.12 MPa. It is worth noting, however, that TBD and DBU alone used as catalysts also gave very good results (over 92% yield and over 97% selectivity), even at a lower temperature (50 °C), but with a much longer reaction time (20 h). As shown in Figure 4, according to the authors, DBU attacks the carbon atom of the epoxide ring, and the resulting intermediate I reacts with carbon dioxide.

One year later, Wu et al. demonstrated DESs containing choline chloride and poly(ethylene glycol) (PEG) of various chain lengths (PEG200–PEG1000) [28]. PEG is a thermally stable, non-toxic, cheap and widely used polymer. The researchers pointed out that PEGs in different catalytic systems had already been used to catalyze the reactions of CO_2_ with epoxides with good results [56,57]. The addition of PEG diminishes the viscosity of the liquid phase and facilitates the diffusion between gaseous and liquid phases, which in turn can enhance the reaction rate due to better CO_2_ adsorption. In the DESs used in this study, PEG plays the role of a hydrogen bond donor. The researchers evidenced that the activity of the studied DES was dependent on the length of the PEG chain. The longer the chain, the lower the yield of cyclic carbonates. It has been suggested that this may be due to mass transport limitations. Additionally, the CO_2_ solubility was different in DESs consisting of different PEGs. The best results (99.1% yield of propylene carbonate) were obtained for ChCl:PEG200 DES in a molar ratio of 1:2, at 150 °C and 0.8 MPa for 5 h without any additional co-catalysts or solvents. Distillation was used to separate the post-reaction mixture. The recovered catalyst was used in subsequent cycles. After the fifth cycle, a drop in yield from 99.1 to 95.6% was observed with a negligible decrease in selectivity.

**Figure 5:**
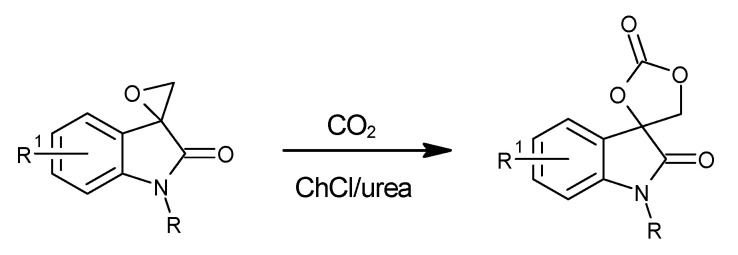
Structure of DES obtained from choline chloride and NBS in a molar ratio of 1:2. R^1^ = CH_3_, CH_3_O, F; R = CH_3_, C_4_H_9_, CH_2_=CHCH_2_.

In 2018, Tak et al. applied a DES-based catalytic system for the synthesis of spiro-cyclic carbonates by the cycloaddition of CO_2_ to spiro-epoxy oxindoles [58]. There was no need for using any additional co-catalyst and the reaction conditions were fairly benign. The model reaction was the cycloaddition of carbon dioxide to *n*-benzyl spiro-epoxyoxindole to obtain the corresponding carbonate (Figure 5). All tested deep eutectic solvents were based on choline chloride with different HBDs, such as glycerol, urea, benzoic acid and ethylene glycol. The best result (98% yield) was achieved while using ChCl:urea DES (similarly as in the work by Zhu et al. [50]) with a reaction time equal to only 2 h, at a temperature of 70 °C and under atmospheric pressure. In addition to this, the used catalyst could be separated and then reused in at least the next four runs. However, the conducted separation process was more complicated and less environmentally friendly than the methods documented in previous works. 2-Methyl tetrahydrofuran and water were added to the mixture and stirred, and then the water phase was separated and washed three times with 2-methyl tetrahydrofuran. Finally, the water was evaporated and the DES could be recovered.

**Figure 6:**
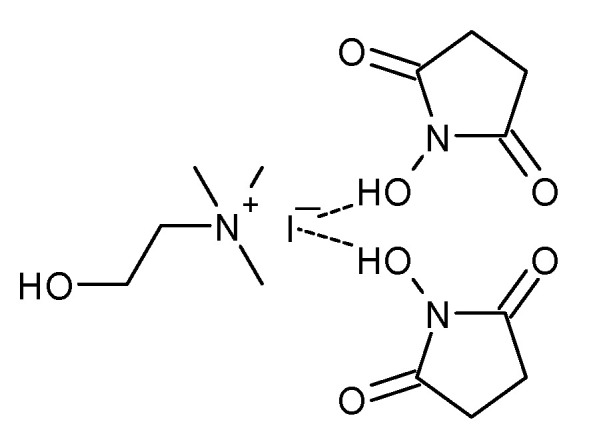
Structure of DES obtained from choline chloride and NBS in a molar ratio of 1:2.

In 2019, Liu et al. screened a series of DES based on *N*-hydroxysuccinimide (NHS) and choline halides (Figure 6) for the synthesis of cyclic carbonates from epoxides and CO_2_ [43]. Such DESs proved to be highly effective catalysts. For example, DES obtained from choline iodide (ChI) and NHS in a molar ratio of 1:2 was the best catalyst for the reaction of propylene oxide and CO_2_, affording PC in high yield (96%) and selectivity (99%) at 30 °C, and 1.0 MPa pressure, after 10 h. However, the reaction time can be reduced to 1 h if the temperature is increased to 60 °C, which allows a PC to be obtained in 92% yield with 99% selectivity. The catalyst can be easily recovered by extraction with ether and reused for at least in five consecutive runs without any significant loss in activity. In comparison to previous works on the use of deep eutectic solvents in the reaction of cycloaddition of CO_2_ to epoxides, DESs based on NHS exhibit higher activity and made significant progress in terms of carrying out this synthesis under ambient conditions.

Soon after, the same researchers tested a series of DESs comprising tetra-*n*-butylphosphonium bromide (TBPB) as HBA and different aromatic substrates (phenol, aminophenol (AP), aniline and benzoic acid) as HBDs in the synthesis of cyclic carbonates from CO_2_ and epoxides [40]. They found that the TBPB:3-AP deep eutectic solvent was the most excellent catalyst, giving propylene carbonate in high yield (99%) and selectivity (96%) in fairly mild reaction conditions (80 °C, 1 MPa and only 1 h reaction time). A lower reaction temperature with a longer reaction time was also tested and gave results that were nearly as good. At a temperature of 30 °C after 24 h, the yield was slightly lower (95%) and the selectivity remained at the same level. Additionally, DESs with benzoic acid and 2- and 4-AP gave yields over 90% and selectivity over 99%. In addition, the catalyst could be recovered by solvent extraction and used for at least 5 consecutive cycles without significant loss in selectivity, although with a slight drop in yield. TBPB:3-AP as a catalyst was also applied in the synthesis of cyclic carbonates from different epoxides and gave favourable results as well (99–87%), except for the cyclohexene oxide (19% yield), where the steric hindrance probably impeded the epoxide ring opening and the CO_2_ molecule incorporation. The researchers pointed out that phosphonium-based deep eutectic solvents could be such efficient catalysts for cyclic carbonate formation because of their Lewis basicity, nucleophilicity, and the ability to create hydrogen bonds.

Subsequently, Vagnoni et al. developed DESs based on choline chloride and iodide with different hydrogen bond donors such as polyols and carboxylic acids [36]. One of the goals of the work was to synthesize active DES from cheap and naturally derived HBDs. The model reaction was the synthesis of styrene carbonate (SC) from styrene oxide (SO) and CO_2_. Experiments involving choline chloride-based DES were carried out at a pressure of 0.4 MPa at a temperature of 80 °C for 8 h. The highest yield (97%) was achieved for three different ChCl-based DESs: with maleic acid (selectivity 98%), malonic acid (selectivity 99%), and malic acid (selectivity also 99%). When the reaction was carried out under atmospheric pressure, SO conversion was not high (maximum 26%) but the selectivity reached 98%. The researchers compared the reactions conducted with DESs prepared before the process with those in which HBD and HBA were introduced separately into the reactor. Interestingly, some of the in situ generated DESs gave similar results to the preformed ones. For example, for DES based on ChCl and ethylene glycol, the PC yield was 82%, and for the components introduced separately, it was 80%, while in the other cases for previously prepared DES, the yield was higher by 12 (for ChCl:tartaric acid) or 21 percentage points (for ChCl:glycerol). DESs containing choline iodide were also tested. In this case, the DES components were added separately to the reaction mixture. The best outcome was reached for the mixture with glycerol in the reaction carried out 80 °C and under atmospheric pressure for 7 h. The SO conversion was 99% and the selectivity to SC was 96%. Such good results are due to the halide, which is responsible for opening the epoxide ring and is able to form hydrogen bonds that can stabilize intermediates. The catalyst can be recovered by extraction with water. After evaporation of the water, without further purification, the catalyst was ready for reuse for at least four more cycles, while maintaining its activity.

An interesting study was presented by Xiong et al. in 2020 [51]. As a catalyst for the cycloaddition reaction of CO_2_ to epoxides, the group applied a deep eutectic solvent consisting of choline chloride and *p*-aminobenzoic acid (PABA) immobilized on lignin, together with TBAB as a co-catalyst. The model reaction was the cycloaddition of CO_2_ to phenyl glycidyl ether. Under the most favourable conditions (110 °C, 1.0 MPa, 3 h), the product was obtained with a yield of 99%. Nevertheless, it was necessary to add TBAB in an amount of 10 mol% relative to the epoxide. The same catalytic system was also tested in the reactions with other epoxides, affording the corresponding products in high yields (90–99%) and selectivity (over 90%). It was suggested that hydroxyl and amino groups on the surface of the catalyst were responsible for such good performance of this catalytic system. The applied heterogeneous catalyst exhibited high activity and stability and could be simply recycled by filtration and then reused in five consecutive runs, during which the selectivity remained on the same level (99%), while the yield dropped from 99 to 84%. Scaling up of the reaction was also investigated. As the scale increased, the product yield decreased, but even at an 8-fold scale-up, the product yield was greater than 90%. However, the disadvantage of this method is the relatively high reaction temperature. Other drawbacks are the necessity of using the co-catalyst and the multistep time-consuming catalyst preparation procedure.

**Figure 7:**
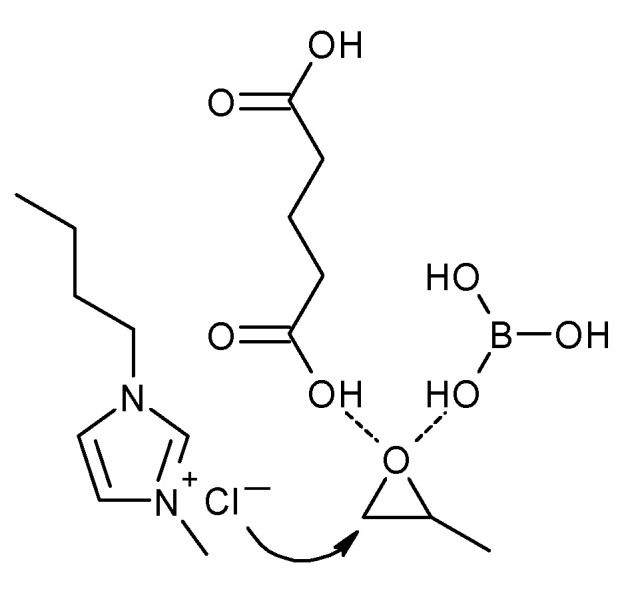
Activation of the epoxy ring by DES based on bmimCl, BA, and GA.

In 2020, Wang et al. developed an innovative type of deep eutectic solvent for the synthesis of cyclic carbonates [32]. The group reported the excellent performance of ternary DESs in the synthesis of these compounds from carbon dioxide and different epoxides. The researchers wanted to avoid the use of iodide and bromide anions due to their negative environmental impact. The best performance was achieved for the composition of 1-butyl-3-methylimidazolium chloride (bmimCl), boric acid (BA), and glutaric acid (GA) in a 7:1:1 molar ratio. The propylene carbonate yield reached 98.3% at the temperature of 70 °C, under a pressure of 0.8 MPa after 7 h. The catalytic performance of this DES was compared to the classic binary systems containing bmimCl:BA and bmimCl:GA. It was demonstrated that the ternary system was superior. The suggested explanation for this occurrence was the acidity of the applied catalysts, as the ternary system showed the highest acidity and bmimCl:BA the lowest. Strong acidity of DES promotes the formation of hydrogen bonds between DES and oxygen of the epoxy ring of PO, which activate the PO molecule (Figure 7). The ternary DES was also applied for the synthesis of other cyclic carbonates, affording very good results (yields: 87.9–99.0%), except for the cyclohexene oxide (39.5%). The catalyst could also be easily separated after the reaction and reused in the next run; however, the product yield decreased by about 20% after 5 consecutive runs.

**Figure 8:**
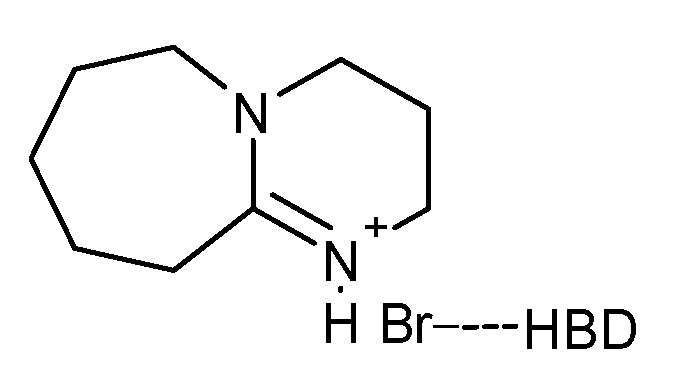
DES based on [DBUH][Br] and HBD.

Even greater progress was demonstrated by Yang et al. in their 2021 paper [23]. Processes presented in their work seem to be almost flawless in terms of reaction conditions. They succeeded in applying the appropriate DES in the synthesis of cycloaddition of CO_2_ to epoxides at room temperature and under atmospheric pressure, achieving product yields of up to 99%. However, the required synthesis time was 48 h. It is worth noting that no extra solvents or additives were used and the catalyst could be recycled and used in the next five consecutive runs without loss in activity. The researchers synthesized a deep eutectic solvent from protic ionic liquids and amines (Figure 8). It was assumed that such a catalytic system could give a great outcome, as amines can absorb carbon dioxide and act as an HBD. Additionally, protic ionic liquids have already been successfully applied as catalysts in the synthesis of cyclic carbonates [59,60]. DES consisting of these two components could potentially increase the concentration of CO_2_ in the liquid phase and activate the carbon dioxide and epoxide molecules. It transpired that this assumption was right. After carrying out the reactions and investigating their mechanisms, it was confirmed that the high catalytic activity of the studied DESs was caused by synergistic catalysis of protic IL and amine.

The model reaction was the synthesis of styrene carbonate from styrene oxide and CO_2_. The reaction was carried out under ambient conditions; however, the time was quite long (48 h). The best results were obtained for two different deep eutectic solvents: [DBUH][Br]/DEA (1,8-diazabicyclo[5.4.0]undec-7-ene bromide and diethanolamine) and [TMGH][Br]/DEA (1,1,3,3-tetramethylguanidine bromide and diethanolamine). In both cases, the yield reached 97% and the selectivity was more than 99%. Under the best conditions (25 °C, atmospheric pressure, 48 h), a series of cyclic carbonates was obtained with yields ranging from 94 to 99%. For [DBUH][Br]/DEA, the synthesis was also carried out at a temperature of 60 °C, which allowed a decrease in the reaction time to 5 h. The main drawback of this method is the significant amount of the catalyst that should be used in this process—20 mol%. Moreover, the DES components might be expensive. The influence of water on the reaction was also investigated, and it was shown that its addition negatively affects the reaction yield. This creates the necessity of drying the reagents before applying them to the reaction.

In 2021, Liu et al. pointed out that in many previous studies regarding deep eutectic solvents as catalysts in the cycloaddition reaction of CO_2_ to epoxides, the DES incorporated the HBA with a nucleophilic site (usually halogen anion) and the HBD with Brønsted acidic site (active hydrogen) [49]. The group came up with the idea of creating a DES in which both the HBA and the HBD have Brønsted acidic sites, and thus the epoxide could be activated more efficiently. They chose to use imidazolium salts as HBAs, as it had already been proven that they could activate epoxides because of their Brønsted acidic site, and different aromatic compounds with active hydrogens as HBDs (e.g., benzoic acid, aniline, o-aminophenol, dihydroxybenzenes). Indeed, such a catalyst gave a great performance in the synthesis of styrene carbonate from styrene oxide and carbon dioxide. At room temperature, atmospheric pressure and a reaction time of 12 h, 97% yield and over 99% selectivity could be achieved using a DES consisting of 1-ethyl-3-methylimidazolium iodide (emimI) and *m*-dihydroxybenzene (*m*-DHB). The catalytic system was also tested for other epoxides, and each time (except for cyclohexene oxide) the yield reached 98 or 99%. The catalyst was separated from the reaction mixture by extraction with ethyl acetate and ether and then tested in five consecutive runs. The selectivity remained at the same level (>99%), while the yield decreased from 97% to 79%. It was suggested that this drop in yield may be due to the loss of the catalyst during its recovery process. The main advantage of this method is the mild synthesis conditions, while the drawbacks may still be a relatively long reaction time and the DES components—both are hazardous and toxic, which creates problems with their maintenance and disposal.

He et al. published a paper in which DESs based on compounds derived from biomass (biomass-derived deep eutectic solvents, BDESs) were used as catalysts for the synthesis of cyclic carbonates from epoxides and carbon dioxide [24]. It is an important issue, as the significance of biomass and its products is increasing in the chemical industry. Biomass is a virtually unlimited source of basic chemicals, which can be converted into valuable and highly desirable compounds. Biomass is an eco-friendly alternative for these kinds of substances, usually originating from fossil resources. The list of chemicals that can be obtained from biomass is very long and includes many compounds that can be used to synthesize deep eutectic solvents, such as malic acid, levulinic acid, glycerol or xylitol [61]. He et al. [24] used choline chloride, iodide and bromide as HBAs, which were mixed with different HBDs—citric acid, 3-methylglutaric acid, levulinic acid and 2-hydroxypropionic acid. The reaction of propylene oxide with CO_2_ was carried out for 3 h, at a temperature of 70 °C, under a pressure of 1.0 MPa. The best results were achieved using DES obtained from choline iodide and citric acid; the reaction yield was 98% and the selectivity to propylene carbonate was over 99%. BDESs are highly stable substances, and despite being homogenous catalysts, they can be easily recovered and reused in the next five runs without relevant loss of activity. To recover the catalyst, ethyl acetate was added to the post-reaction mixture to extract the BDES, and after evaporation of the solvent, they were dried under vacuum (BDESs are highly soluble in polar solvents such as methanol, ethanol and water).

A similar study was carried out by Yang et al. [22]. They focused on bio-based deep eutectic solvents and their use in the reaction of CO_2_ with epoxides leading to cyclic carbonates. This time, they investigated deep eutectic solvents based on choline bromide and also on acetylcholine bromide (AcChBr) as HBAs. Malonic acid, L-malic acid, succinic acid and glycerol were applied as HBDs. The model reaction of styrene oxide with CO_2_ was carried out under atmospheric pressure at a temperature of 80 °C for 2 h. The authors also applied less of the catalyst than in their previous research—10 mol%. The best results were obtained using DES consisting of acetylcholine bromide and L-malic acid. The product yield was 98% and the selectivity reached over 99%. The catalyst could be easily separated and used in the next five runs without losing its activity. The studied DES also transpired to be very stable—comparing FTIR spectra of the freshly prepared catalyst and the one used in five cycles, there was no significant difference in their structures.

Very recently, TBAB-based DESs were used as catalysts for cyclic carbonate synthesis [62]. Wang et al. paid attention to the use of hydroxyl groups in the catalyst, as they can positively affect the reaction of hydrogen bonds. It was assumed that hydroxyl groups could potentially enhance the epoxide activation and have the ability to stabilize the intermediate. Therefore, as HBDs, glycerol, 1,3-propanediol (PG), malonic acid and citric acid (CA), and as HBAs, tetrabutylammonium salts with Cl, Br and F anions, were applied. In the model reaction between PO and CO_2_, the most active was the DES composed of CA and TBAB in a molar ratio of 1:2.5. Reactions were carried out at a temperature of 80 °C, under a pressure of 0.8 MPa for 1 h. After the reaction, ethyl acetate and water (5:1 *v*/*v*) were added to the reaction mixture. Then, the separated aqueous layer was dried, and the recovered catalyst could be used for the next reaction. The recycled catalyst retained its selectivity in the next 5 cycles; however, during this time the yield diminished by about 5 percentage points.

**Figure 9:**
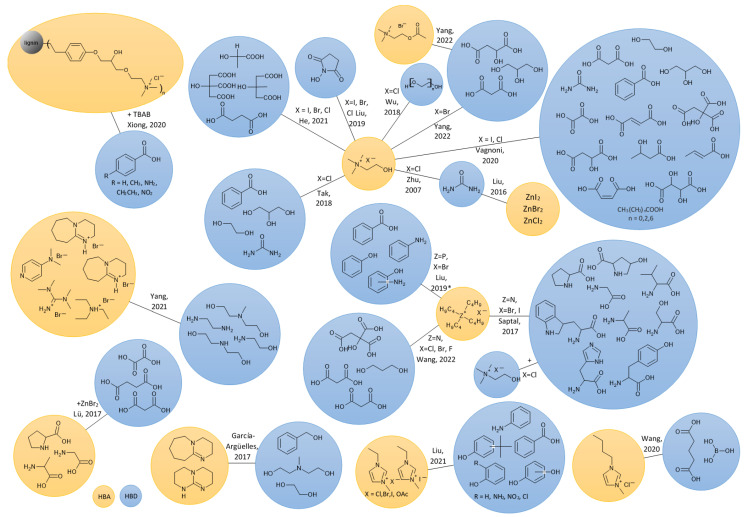
Various DESs used in the synthesis of cyclic carbonates from CO_2_ and epoxides. Zhu, 2007 [50]; Liu, 2016 [46]; García-Argüelles, 2017 [53]; Lü, 2017 [33]; Saptal, 2017 [52]; Tak, 2018 [58]; Wu, 2018 [28]; Liu, 2019 [43]; Liu, 2019 * [40]; Wang, 2020 [32]; Vagnoni, 2020 [36]; Xiong, 2020 [51]; He, 2021 [24]; Liu, 2021 [49]; Yang, 2021 [23]; Wang, 2022 [62]; Yang, 2022 [22].

Table 1 presents examples of results obtained under the best-established conditions for the synthesis of cyclic carbonates from CO_2_ and epoxides using DESs as catalysts, while Figure 9 presents a summary of the structures of various DES used in this process. Turnover number (TON) and turnover frequency (TOF) values presented in Table 1 in most cases range from 4.6 to 49.1 and from 0.1 to 27.2, respectively. The highest TON values were obtained for the DES composed of TBAB and citric acid (102.3), ChCl/urea (95.0–99.0), TBD/BA (98.0) and [urea-Zn]I_2_ (81.7). In all these cases, the reaction temperatures were above 80 °C. The highest TOF showed TBAB and citric acid (102.3) and TBD/BA (49.0) catalytic systems.

## 3. Conclusions

The amount of literature regarding the use of deep eutectic solvents in the synthesis of cyclic carbonates from CO_2_ and epoxides increases every year. The main reasons to apply DESs in the cycloaddition of CO_2_ to epoxides, among a variety of other different catalysts, are the simplicity of preparation, often simple and widely available starting materials, low price, stability and biodegradability. DESs also have the ability to capture carbon dioxide [63], which may positively affect the reaction, as CO_2_ is one of the two main substrates.

The most commonly used deep eutectic solvents for the synthesis of cyclic carbonates, which give the best results, are the compositions based on choline halides as hydrogen bond acceptors. Other HBAs already applied are acetylcholine halides, quaternary ammonium halides, amino acids (such as L-proline), and protic ionic liquids. An even greater variety of compounds can be observed among the hydrogen bond donors. Common HBDs include carboxylic acids (e.g., oxalic acid, malonic acid, malic acid, levulinic acid), amines (such as diethanolamine), amides (e.g., urea), imides (e.g., *N*-hydroxysuccinimide), polyols (e.g., glycerol, ethylene glycol) and even polymers (the example of DESs with poly(ethylene glycol)).

Regarding the mechanism of the studied synthesis, it is currently believed that the formation of hydrogen bonds between the catalyst components and the epoxide, as well as the additional interaction of the epoxide with a nucleophile (usually halides), are of major significance. This way, the epoxy ring is opened and the CO_2_ molecule can be incorporated. After this, the ring closes back and the catalyst is regenerated.

Currently, the main goals in the cyclic carbonate synthesis involving DESs are to reduce the reaction temperature and pressure to the ambient values, not to use any co-catalysts or additional agents (especially those containing metals), to reduce the amount of the catalyst and to use compounds that are benign and renewable, while achieving maximum activities and yields. Shortening the reaction time is also important, as well as the easiness of the catalyst and product separation after the reaction and purity of the product. Particular attention is paid to sustainable and eco-friendly deep eutectic solvents comprised of natural or bio-based substrates, especially derived from biomass. All these activities contribute to the development of energy-saving, material-saving, and environmentally safe methods for the synthesis of cyclic carbonates.

## Figures and Tables

**Table 1:** Comparison of the results of cyclic carbonate synthesis with various DES-based catalysts.

DES		HBA:HBD Molar Ratio	DES, mol%	Epoxide	Reaction Parameters			Yield, %	Selectivity, %	TON ^1^	TOF ^2^, h^−1^	Ref.
HBA	HBD				Temperature, °C	Pressure, MPa	Time, h					
ChCl	urea	1:2	1.0	PO	110	- ^3^ - ^4^	10 4	99 99 ^5^	>99	95.0 99.0	9.5 24.8	[50]
ZnI_2_	urea	1:3	1.2	PO	120	1.5	3	95	98	81.7	27.2	[46]
TBAI	[Ch][His]	1:1	20.0	EP ^6^	70	0.1 1.0	30 2	92 ^7^ 98 ^7^	99 99	4.6 4.9	0.2 2.5	[52]
L-Pro + ZnBr_2_	propanedioic acid	1:2	2.0	PO	150	1.2	5	98	-	49.1	9.8	[33]
TBD	benzyl alcohol	1:1	1.0	EP	100	0.1	2	98	98	98.0	49.0	[53]
ChCl	PEG200	1:2	2.1	PO	150	0.8	5	99	>99	47.2	9.4	[28]
ChCl	urea	1:2	577.5	*n*-benzyl spiro-epoxyoxindole	70	0.1	2	98	-	0.2	0.1	[58]
ChI	NHS	1:2	6.0	PO	30 60	1.0	10 1	96 92	99 99	16.0 15.3	1.6 15.3	[43]
TBPB	3-AP	1:2	4.5	PO	30 80	1.0	24 1	95 96	>99 >99	21.1 21.3	0.9 21.3	[40]
ChI	glycerol	1:1	5.0	SO	80	0.1	7	90	-	18.0	2.6	[36]
ChCl	PABA ^8^	1:1	10.0	phenyl glycidyl ether	110	1.0	3	99	>99	9.9	3.3	[51]
bmimCl	GA + BA ^9^	7:1:1	7.0	PO	70	0.8	7	98	-	14.0	2.0	[32]
[DBUH][Br]	diethanoloamine	2:1	20.0	SO	r.t ^10^ 60	0.1	48 5	97 >99	>99	4.9 5.0	0.1 1.0	[23]
emimI	*m*-DHB ^11^	2:1	10.0	SO	r.t.	0.1	24	99	>99	9.9	0.4	[49]
ChI	citric acid	2:1	3.0	PO	70	0.5	3	98	>99	32.7	10.9	[24]
AcChBr	L-malic acid	2:1	10.0	SO	80	0.1	2	98	>99	9.8	4.9	[22]
TBAB	citric acid	2.5:1	0.9	PO	80	0.8	1	95	99	102.3	102.3	[62]

^1^ moles of cyclic carbonate produced per mole of DES-based catalyst. ^2^ moles of cyclic carbonate produced per mole of DES-based catalyst per hour. ^3^ molar ratio of CO_2_ to epoxide 2.45. ^4^ molar ratio of CO_2_ to epoxide in the range of 1.5–1.87. ^5^ DES immobilized on molecular sieves. ^6^ epichlorhydrin. ^7^ conversion of EP. ^8^ *p*-aminobenzoic acid (PABA); DESs-modified lignin heterogeneous catalysts. ^9^ boric acid (BA), glutaric acid (GA). ^10^ room temperature. ^11^ *m*-dihydroxybenzene.

## Data Availability

Not applicable.
